# Comparison of dynamic and static spacers for the treatment of infections following total knee replacement: a systematic review and meta-analysis

**DOI:** 10.1186/s13018-022-03238-7

**Published:** 2022-07-15

**Authors:** Jiasheng Tao, Zijian Yan, Bin Pu, Ming Chen, Xiaorong Hu, Hang Dong

**Affiliations:** 1 The First Clinical Medical College, Guangzhou University of Chinese Medicine, Number12, Jichang Road, Baiyun District, Guangzhou, 510405 Guangdong Province China; 2 Department of TCM Orthopedics, Hospital for First Affiliated Hospital of Guangzhou University of Chinese Medicine, Number16, Jichang Road, Baiyun District, Guangzhou, 510405 Guangdong Province China

**Keywords:** Static spacers, Dynamic spacers, Periprosthetic joint infection, Total knee arthroplasty, Meta-analysis

## Abstract

**Background:**

Revision surgery is the most common treatment for patients who develop infection after total knee arthroplasty (TKA). Two types of spacers are often used in revision surgery: dynamic spacers and static spacers. The comparative efficacy of these two types of spacers on knee prosthesis infections is not well established. Therefore, we carried out a systematic evaluation and meta-analysis with the aim of comparing the difference in efficacy between dynamic and static spacers.

**Methods:**

We conducted the literature search in PubMed, Web of Science, Cochrane Library, and Embase databases. The articles searched were clinical study comparing the difference in efficacy between dynamic spacers and static spacers for the treatment of prosthetic infections occurring after total knee arthroplasty.

**Results:**

We conducted a literature search and screening based on the principles of PICOS. Ultimately, 14 relevant clinical studies were included in our current study. We use infection control rate as the primary evaluation indicator. The KSS knee scores (KSSs), KSS functional scores, bone loss and range of motion (ROM) are secondary indicators of evaluation. Thirteen of these included studies reported the infection control rates, with no significant difference between dynamic and static shims (RR: 1.03; 95% Cl 0.98, 1.09; P = 0.179 > 0.05). The KSSs were reported in 10 articles (RR: 5.98; 95% CI 0.52, 11.43; P = 0.032 < 0.05). Six articles reported the KSS functional scores (RR: 13.90; 95% CI 4.95, 22.85; P = 0.02 < 0.05). Twelve articles reported the ROM (RR: 17.23. 95% CI 10.18, 24.27; P < 0.0001). Six articles reported the bone loss (RR: 2.04; 95% CI 1.11, 3.77; P = 0.022 < 0.05).

**Conclusion:**

Current evidence demonstrates that dynamic spacers are comparable to static spacers in controlling prosthetic joint infection. In terms of improving the functional prognosis of the knee joint, dynamic spacers are more effective than static spacers.

## Background

For patients with severe knee injuries, total knee arthroplasty (TKA) can effectively improve the function of the knee joint, relieve knee pain, and improve the quality of life of patients. Knee prosthesis joint infection (PJI) is one of the most terrible complications after the total knee arthroplasty, and it is often the main reason for the failure of the total knee arthroplasty [1, 2]. The probability of prosthesis joint infection after primary knee replacement varies from 0.4 to 3% [3, 4]. There are many risk factors for PJI in knee prosthesis infection. Age, duration of surgery, diabetes, urinary tract infection and rheumatoid arthritis are all important risk factors for PJI after TKA surgery [5, 6]. Currently, the number of TKA operations worldwide is increasing year by year. However, due to the trend of aging population and rising obesity rate, the incidence of PJI after TKA is also increasing year by year [7, 8].

The diagnosis and treatment of infections in artificial joint prostheses are still challenging, especially in the early stages. The latest diagnosis of knee prosthesis joint infection is largely based on the diagnostic criteria proposed by the Musculoskeletal Infection Society (MSIS), and PJI is diagnosed when one of the following three conditions is met: (1) the presence of a sinus tract that communicates with the prosthesis; (2) pathogens isolated from two or more separate tissue or fluid samples obtained from the affected prosthetic joint; and (3) four of the six criteria identified are met: 1. the elevation of erythrocyte sedimentation rate (ESR) and C-reactive protein (CRP) concentrations; 2. the elevation of white blood cells (WBC); 3. the elevation of the percentage of mesophilic polymorphonuclear leukocytes (PMN); 4. septicemia: septicemia of the affected joint; 5. isolation of microorganisms in cultures in histological analysis at 400 × magnification; 6. more than 5 neutrophils present in each of the 5 high magnification fields [9–11]. Early and definitive diagnosis is the key to treating PJI. Once the diagnosis of PJI is made, the treatment options include conservative treatment with preservation of the joint prosthesis or debridement, and one or two stage prosthetic replacement surgery. The two-stage arthroprosthetic revisions is the gold standard for the treatment of PJI and is the most common treatment in clinical practice. In the tow-stage of prosthetic revision, the infected prosthesis is removed and a joint spacer with antibiotics is placed into the joint to provide continuous anti-infection treatment and then a new prosthesis is reinserted once the infection has been controlled. The two-stage of prosthetic revision surgery has achieved satisfactory results in current clinical practice [12–14].

In the two-stage of the revision surgery, the two types of spacers that are frequently used include dynamic spacers and static spacers [15]. For static spacer, it is easier to design and the production costs of it are cheaper. In addition, static spacers are easier to implant in the joint during surgery. However, the mobility of the patient's knee joint will reduce after static spacer implantation. Static spacers are also prone to a number of complications such as causing bone loss and soft tissue damage [16, 17]. In contrast, dynamic spacers can improve the recovery of patient's knee function and reduce the incidence of associated complications. The use of spacers can provide good flexion and extension after the implantation of a new prosthesis. However, dynamic spacers are more expensive to produce. And they are not as good as static spacers in maintaining joint stability [18, 19]. In terms of infection control, some reports suggest that dynamic spacers and static spacers are similarly effective, while others suggest that dynamic spacers are less effective than static spacers in controlling infection [20–22].

In order to further investigate whether there are any significant differences between dynamic and static spacers for the treatment of knee prosthesis infections in terms of efficacy, complications and functional impact, we conducted a comprehensive search of the literature and collated the data of inclusion literatures, then we did a corresponding meta-analysis and obtained the final results.

## Methods

### Study design and search strategy

**Fig. 1:**
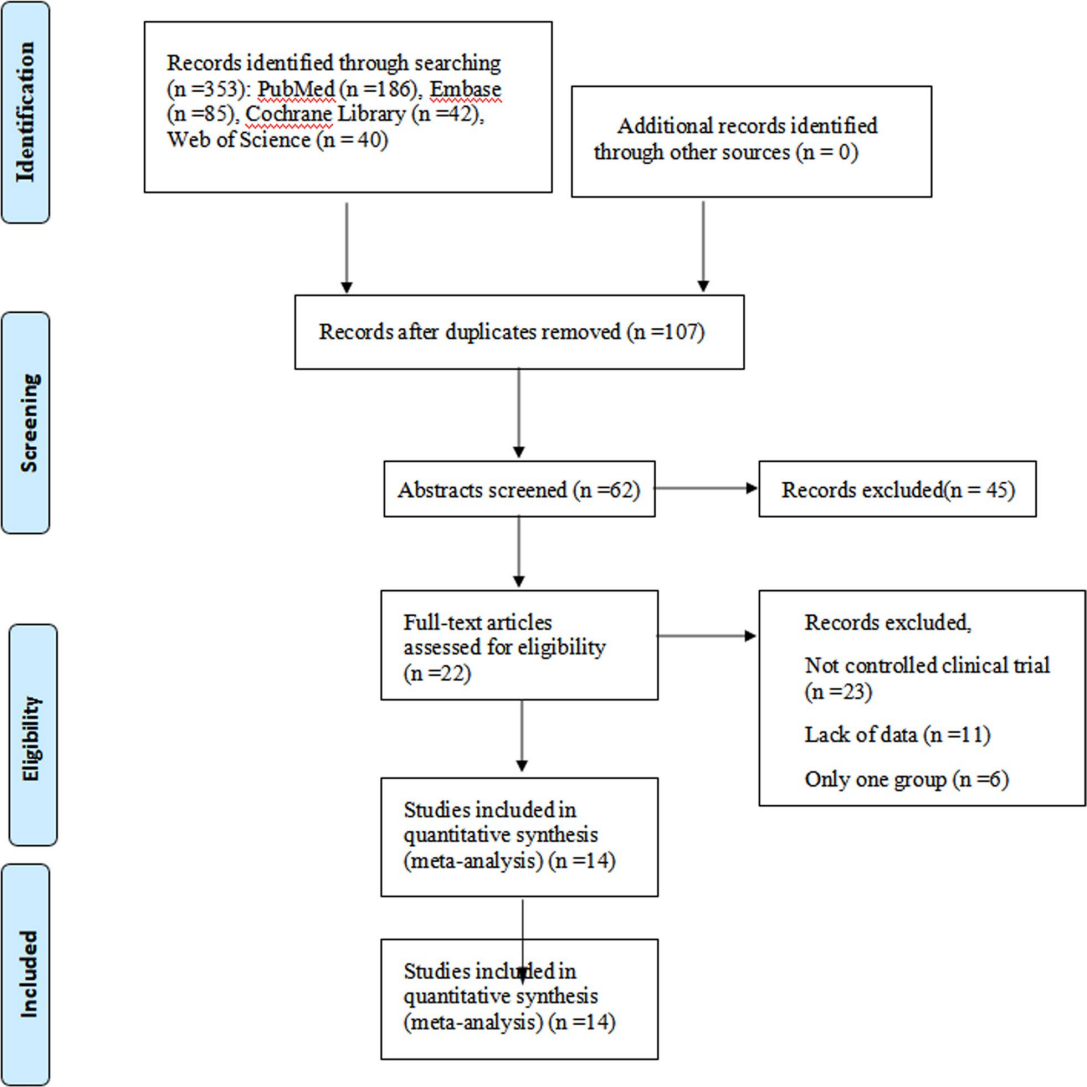
The inclusion process of literature

We followed the principles of The Population-Intervention-Comparators-Outcomes-Study design (PICOS) strictly when conducting our literature search. The databases used for literature searches include PubMed, Embase, Web of Science and Cochrane library databases. The time frame for the search was from database creation to 10 March 2022 and the article type were Clinical randomized controlled studies, retrospective case–control studies or prospective cohort studies of dynamic spacers and static spacers for the treatment of knee prosthesis infections. The following keywords were used in the search process: "static spacer" and "dynamic spacer" and "Knee prosthesis infection", "static spacer" and "Articulating spacer" and "PJI". We had no restrictions on region or population, and all study subjects were human. All patients included in the studies met the diagnostic criteria for joint prosthesis infection as defined in the Infectious Diseases Society of America (IDSA), Musculoskeletal Infection Society (MSIS) [23, 24]. All studies that did not meet this diagnostic criterion were excluded. The specific search process is shown in Fig. 1.

### Inclusion criteria

(I) Clinical randomized controlled studies, retrospective case–control studies or prospective cohort studies; (II) the outcome indicators in the article included one of infection control rate, KSSs score, KSS functional score, ROM or bone loss; (III) complete clinical report; (IV) in the clinical grouping, patients in the trial group used dynamic spacers and patients in the control group used static spacers; (V) there are specific follow-up times in the article and the follow-up times are all over 12 months; (VI) all patients included in the study met the diagnostic criteria for joint prosthesis infection as defined by the Infectious Diseases Society of America (IDSA), Musculoskeletal Infection Society (MSIS).

### Exclusion criteria

In addition to the inclusion criteria described above, we have also developed exclusion criteria. Articles were excluded when one of the following conditions existed: (I) only patients using dynamic spacers or static spacers in the article; (II) there was a lack of follow-up time in the article or the follow-up time was less than 12 months; (III) the outcome indicators and statistical methods associated with the article were not uniform; (IV) literature reviews, case reports or experimental animal studies; (V) the diagnostic criteria for subjects were not referenced to the Infectious Diseases Society of America ( IDSA), Musculoskeletal Infection Society (MSIS).

### Data extraction and management

After determining the final inclusion literatures, we had two researchers responsible for extracting and summarizing the data from the articles, and the inclusion and exclusion of literature strictly followed the criteria described above. Articles were included and excluded by reading the title, abstract and content. If there was a disagreement in the screening and data extraction process, a third researcher was responsible for negotiating a resolution. After the inclusion of literatures, data on infection control rates, KSS scores, KSS functional scores, bone loss, ROM and other relevant indicators were extracted from the literature. We extracted and integrated the data and then performed a meta-analysis.

In addition, after identifying the 14 pieces of literature included, we assessed and scored the methodological quality of each study independently on the Newcastle–Ottawa Scale (NOS) [25]. We evaluated the quality of articles based on the NOS scale for the following items: (I) selection of study population (group), (II) comparability, and (III) how to determine which spacers to use. With a full score of 9 stars, the study is considered to be of high quality when the following conditions exist: (I) 3 or 4 stars in the selection domain, (II) 1 or 2 stars in the comparability domain, and (II) 2 or 3 stars in the outcome or exposure domain. If the NOS score ≥ 7 stars is considered high quality, ≥ 4 and < 7 stars is considered medium quality, and < 4 stars is considered low quality.

### Outcome measurements

Our primary outcome indicator for this meta-analysis was the rate of infection control in the dynamic spacer and static spacer groups. Secondary outcome indicators included KSSs scores, KSS functional scores, bone loss and ROM after treatment with the respective spacers.

### Statistical analysis and data synthesis

After completing the data extraction and classification summary, we used Stata SE-64 to analyze the data and finally obtained the corresponding forest plots for each group. If P < 0.05 indicates that the results are statistically significant and there is a significant difference between the two groups. If P > 0.05 indicates that the results are not statistically significant and there is no difference between the two groups. The variables obtained included dichotomous and continuous variables, and the variables were assessed so that relative risks (RR) and 95% confidence intervals (CI) were used. In terms of article heterogeneity, we used the I^2^ test for analysis. When I^2^ < 50% indicates little heterogeneity between the literature, a fixed effects model (FE) was chosen to analyze the data. When I^2^ > 50% indicates significant heterogeneity between the literature, a random effects model (RE) was chosen to analyze the data.

## Result

### Identification of included studies

After a literature search in PubMed, Embase, Web of Science and Cochrane library databases, a total of 353 relevant articles were initially retrieved. We first read the titles and abstracts of the articles to eliminate duplicates or articles with inconsistent content, leaving 62 articles. The content of the remaining 62 articles was carefully read by two of our researchers, and after excluding 48 of the non-compliant articles, we were left with 14 articles that met the requirements of our study [26–39]. The specific search and selection process for our included articles is referenced in Fig. 1.

### Quality assessment of included studies

We finally included 14 relevant clinical studies. A total of 799 patients were included in all articles, with a total of 407 patients in the treatment group using dynamic spacers and 392 patients in the group using static spacers. The average follow-up time for patients in both treatment groups was more than 12 months. Among the included studies, 9 studies were graded as high quality (≥ 7 stars) and 5 studies were graded as moderate quality (≥ 4 and < 7 stars). The specific characteristics and ratings of each article are shown in Table 1.

**Table 1:** The basic characteristics of the included studies

Study (refs.)	Type of study	Number of participants		Age (years) (**Mean ± SD)**		Intervention				Quality of the literature	Outcomes
		Trial	Control	Trial	Control	Trial	Control	Follow-up time(Trial) (months)	Follow-up time (Control)(months)		
David 2012 [26]	Retrospective	22	14	69	68	DS	SS	20	22	6	KSSs, ROM
Mark 2007 [27]	Retrospective	48	28	67	67	DS	SS	71.2	62.2	7	ICR, KSSs, KSSf
Jessica 2021 [278]	Retrospective	6	10	64.2	64.2	DS	SS	36	84	7	ICR, KSSs, ROM, BL
Thomas 2000 [29]	Retrospective	30	25	NR	NR	DS	SS	27	36	6	ICR, KSSs, ROM, BL
Cindy 2020 [30]	RCT	25	24	65.7	64.9	DS	SS	42	42	9	ICR, KSSs, KSSf, ROM
Park 2009 [31]	Retrospective	16	20	60.2	66.5	DS	SS	29	36	8	ICR, KSSs, KSSf, ROM, BL
Hsu 2007 [32]	Retrospective	21	7	NR	NR	DS	SS	58	101	6	ICR, KSSs, KSSf, ROM
Edward 2022 [33]	Retrospective	104	72	68.6	69.4	DS	SS	120	228	5	ICR, KSSs, KSSf, ROM
Rim 2012 [34]	Retrospective	14	33	64	64	DS	SS	43	63	7	ICR, ROM
Chiang 2011 [35]	Prospective	23	22	71	72	DS	SS	40	40	7	ICR, ROM
Roger 2002 [36]	Retrospective	22	26	65.1	65.7	DS	SS	46	90	6	CEICR, ROM
Aaron 2012 [37]	Retrospective	34	81	62	61	DS	SS	27	66	8	ICR, KSSs, ROM, BL
Jamsen 2006 [38]	Retrospective	22	8	68	70	DS	SS	25.0	48.9	7	ICR, KSSs, KSSf, ROM, BL
Kong 2021 [39]	Retrospective	22	20	67.2	65.5	DS	SS	18	43	8	ICR, KSSs, KSSf, ROM, BL

RCT: Randomized Controlled Trial; NR: not reported; AS: Dynamic Spacer; SS: Static Spacer; ICR: Infection Control Rate; KSSs: KSS knee score; KSSf: KSS function score; ROM: Range of motion; BL: Bone loose

### Infection control rate

**Fig. 2:**
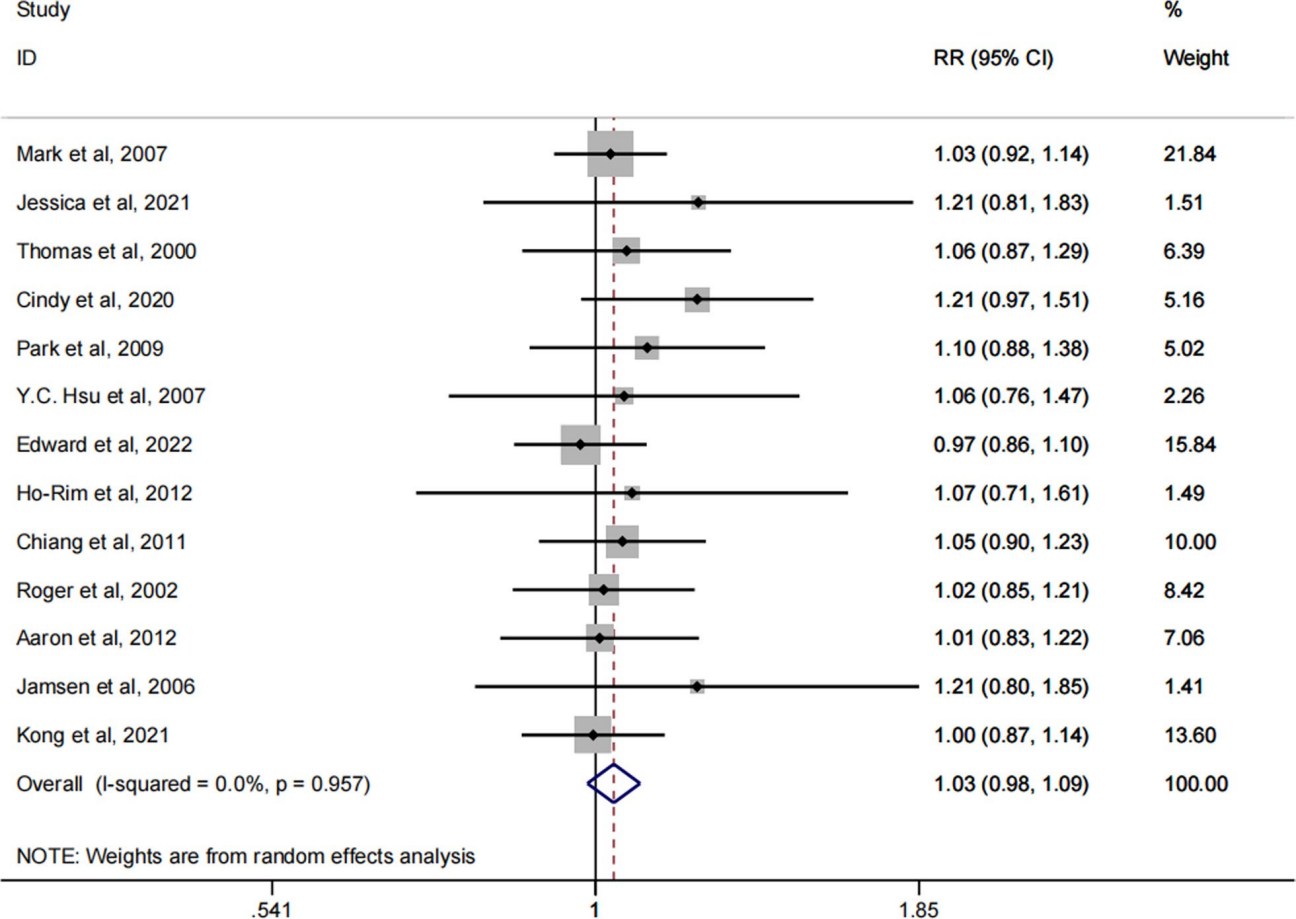
Forest plot of the infection control rate

**Fig. 3:**
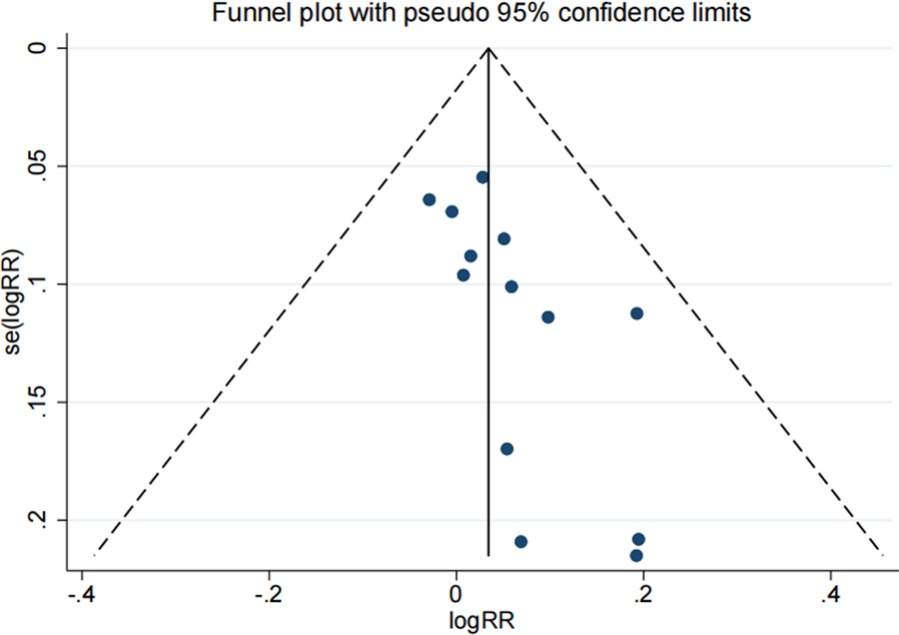
Funnel plot of the infection control rate

The control rate of knee prosthesis infection was the main evaluation index in this meta-analysis. In total, 13 of the 14 included literatures had reported the specific control rates of knee prosthesis infection [27–39]. From the forest plots, we can see that there is no statistical difference between dynamic spacers and static spacers in terms of prosthetic infection control rates. Such results suggest that dynamic spacers and static spacers are similar in controlling prosthetic infection (RR: 1.03; 95% Cl 0.98, 1.09; P = 0.179), as shown in Fig. 2. Such results suggest that dynamic spacers are no less effective in controlling prosthetic infection than static spacers. The funnel plot is shown in Fig. 3. The Egger’s test result is P = 0.015, suggesting some publication bias. For this reason, we assessed this further by using the trim and fill method. And the results remained consistent with those obtained earlier after including the data from five dummy studies. This indicating that the result we derived is steady.

### KSS knee score (KSSs)

**Fig. 4:**
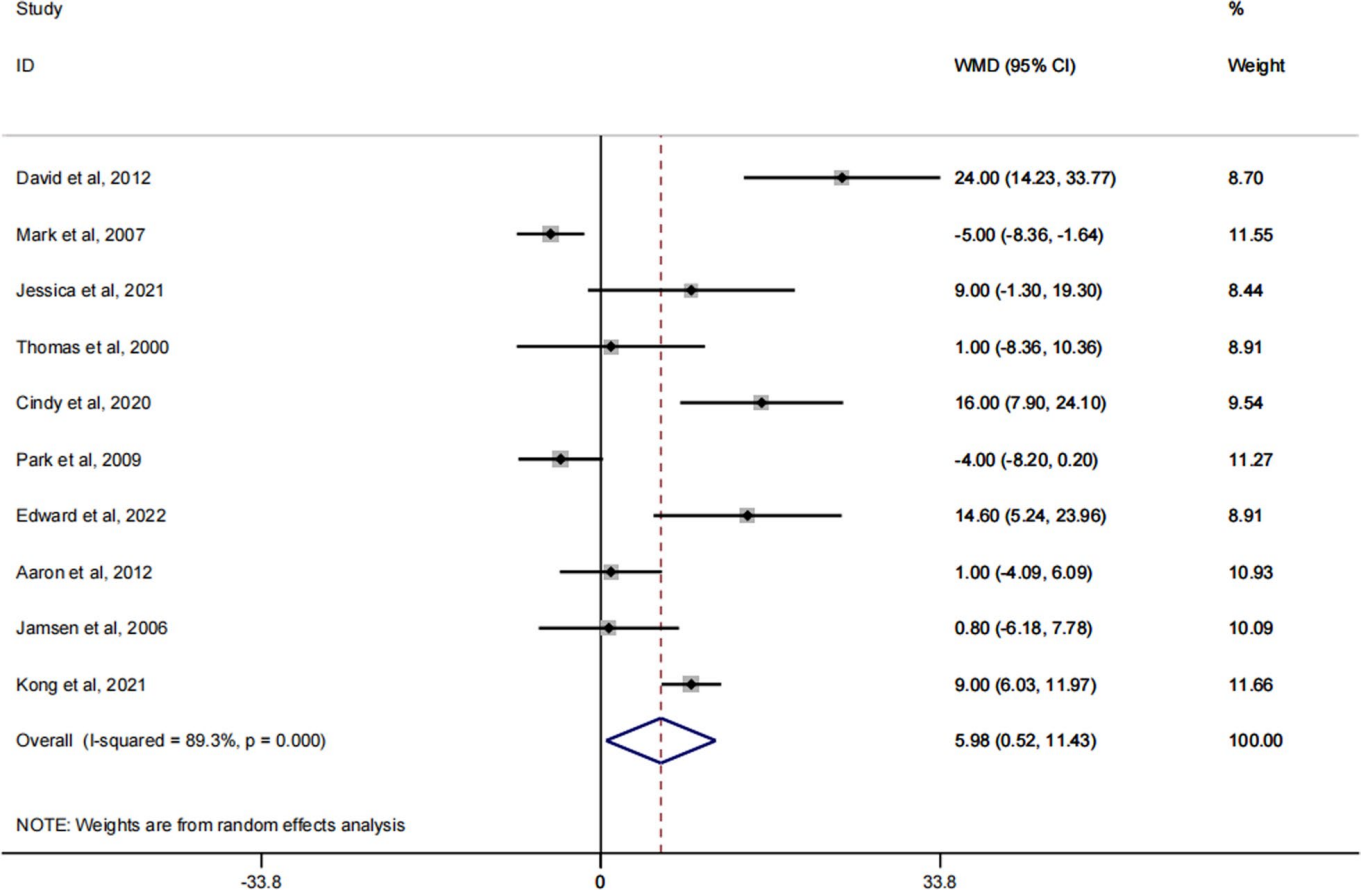
Forest plot of the KSSs

Of the included articles, 10 reported on the KSS Knee Scores (KSSs) of patients after receiving spacer implantation [26–31, 33, 37–39]. Based on the results of the forest plots obtained, it is known that the dynamic spacer was more effective than the static spacer in terms of KSSs scores in the knee joint after spacer implantation, with a statistically significant difference in treatment effect between the two groups (RR: 7.34; 95% CI 1.89, 12.79; p = 0.032). A specific forest plot is shown in Fig. 4.

### KSS functional score

**Fig. 5:**
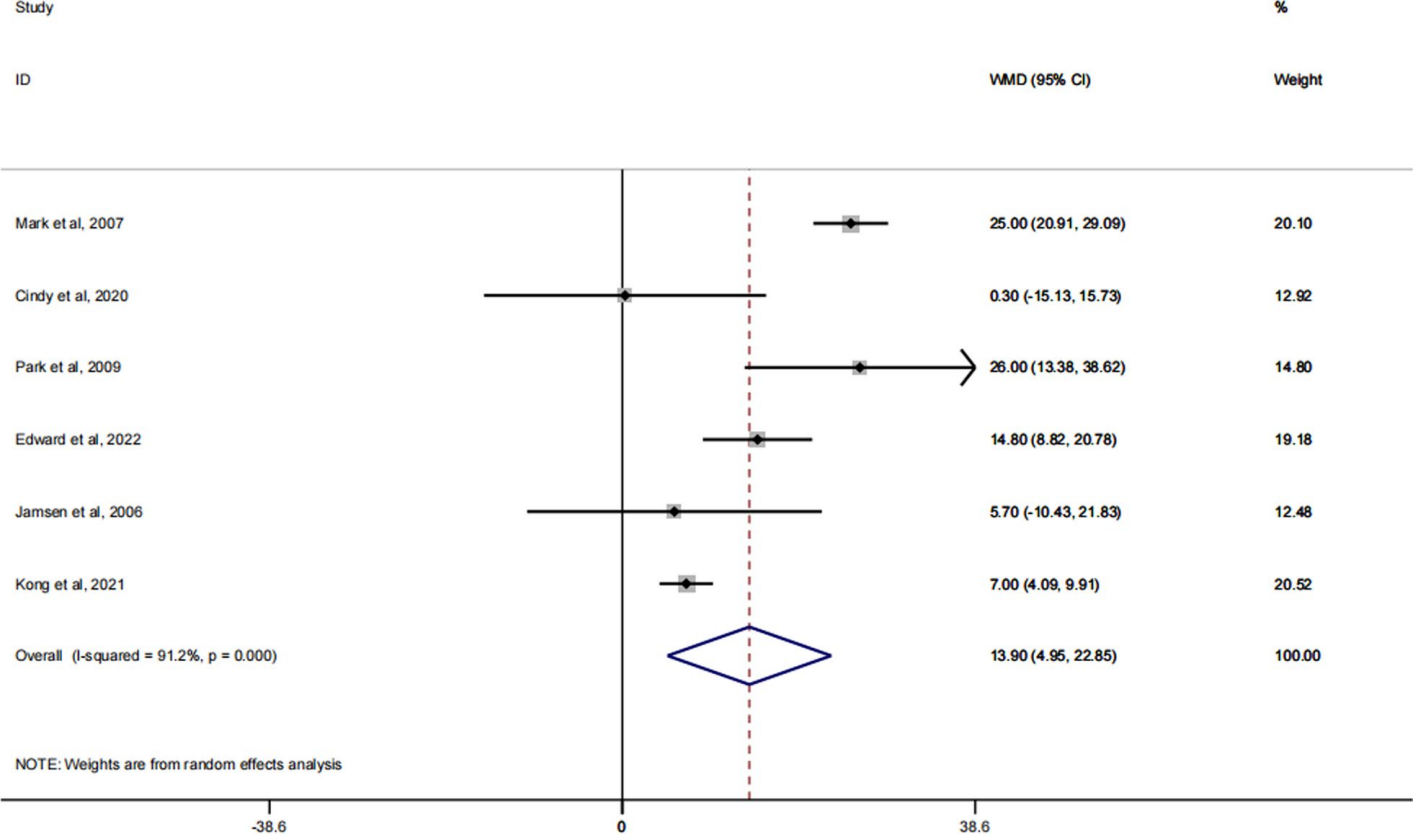
Forest plot of the KSS functional score

Among the secondary outcome indicators in this meta-analysis, the KSS functional score was one of the indicators to judge the effect of the spacer implantation on the patient's later functional recovery of the knee. Of the 14 articles included, a total of 6 articles reported on the KSS functional scores of patients after receiving spacer implantation [27, 30–32, 38, 39]. According to the forest plot we can know that the dynamic spacer was significantly better than the static spacer in improving the patients' recovery of knee function, with statistically significant results between the two (RR: 11.16; 95% CI 4.18, 18.13; P = 0.02). The forest plot is shown in Fig. 5.

### Bone loos

**Fig. 6:**
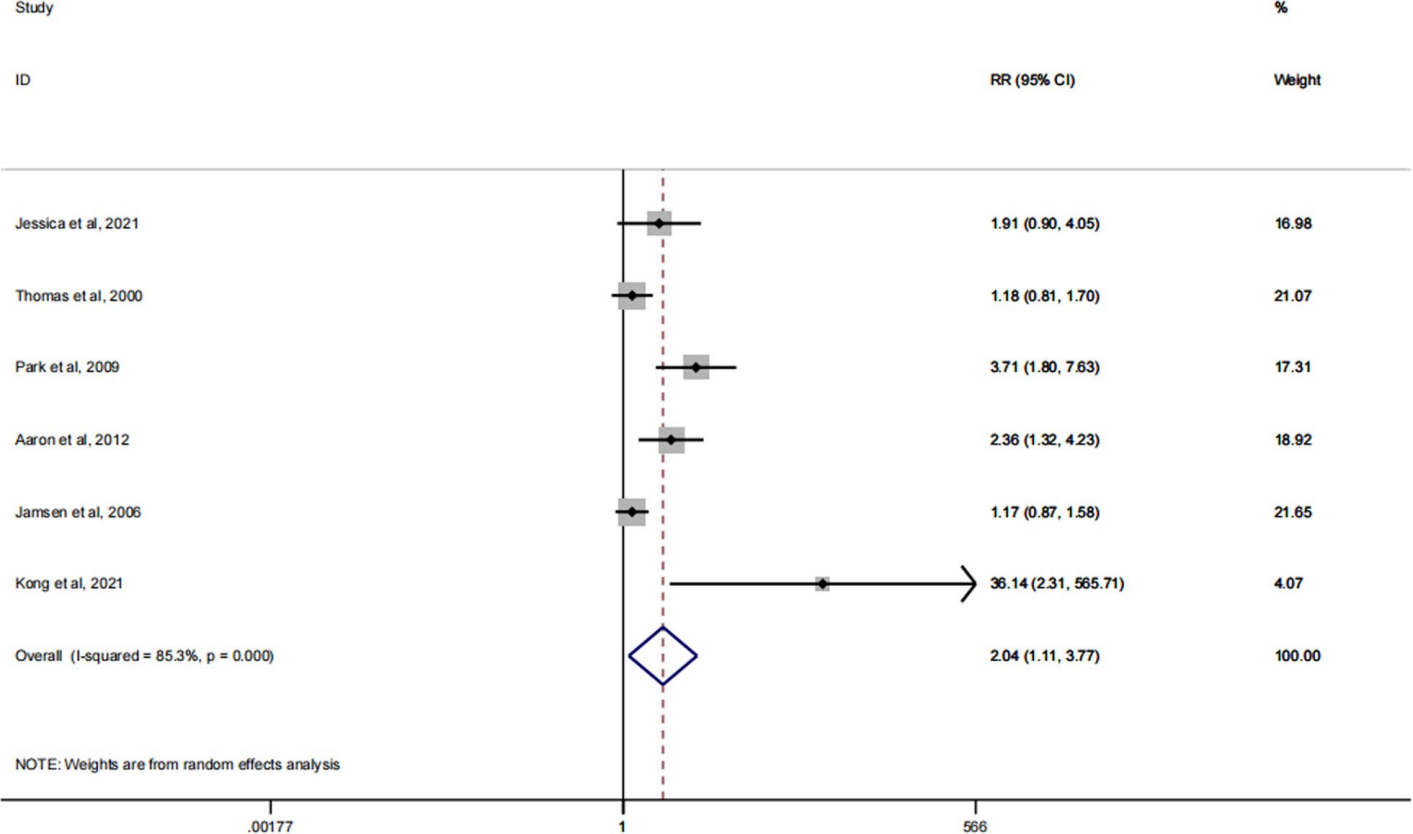
Forest plot of the bone loose

Bone loss is one of the secondary outcome indicators in this meta-analysis, and it is an indicator of the occurrence of bone loss or deficiency in and around the joint after spacer implantation. A total of six articles reported on bone loss after patients received treatment [28, 29, 31, 37–39]. The results suggest that after spacer implantation in our patients, significantly fewer patients with dynamic spacers experienced bone loss than those with static spacers. There was a statistically significant difference between the two groups (RR: 2.04; 95% CI 1.11, 3.77; p = 0.018). The specific forest plot is shown in Fig. 6.

### ROM

**Fig. 7:**
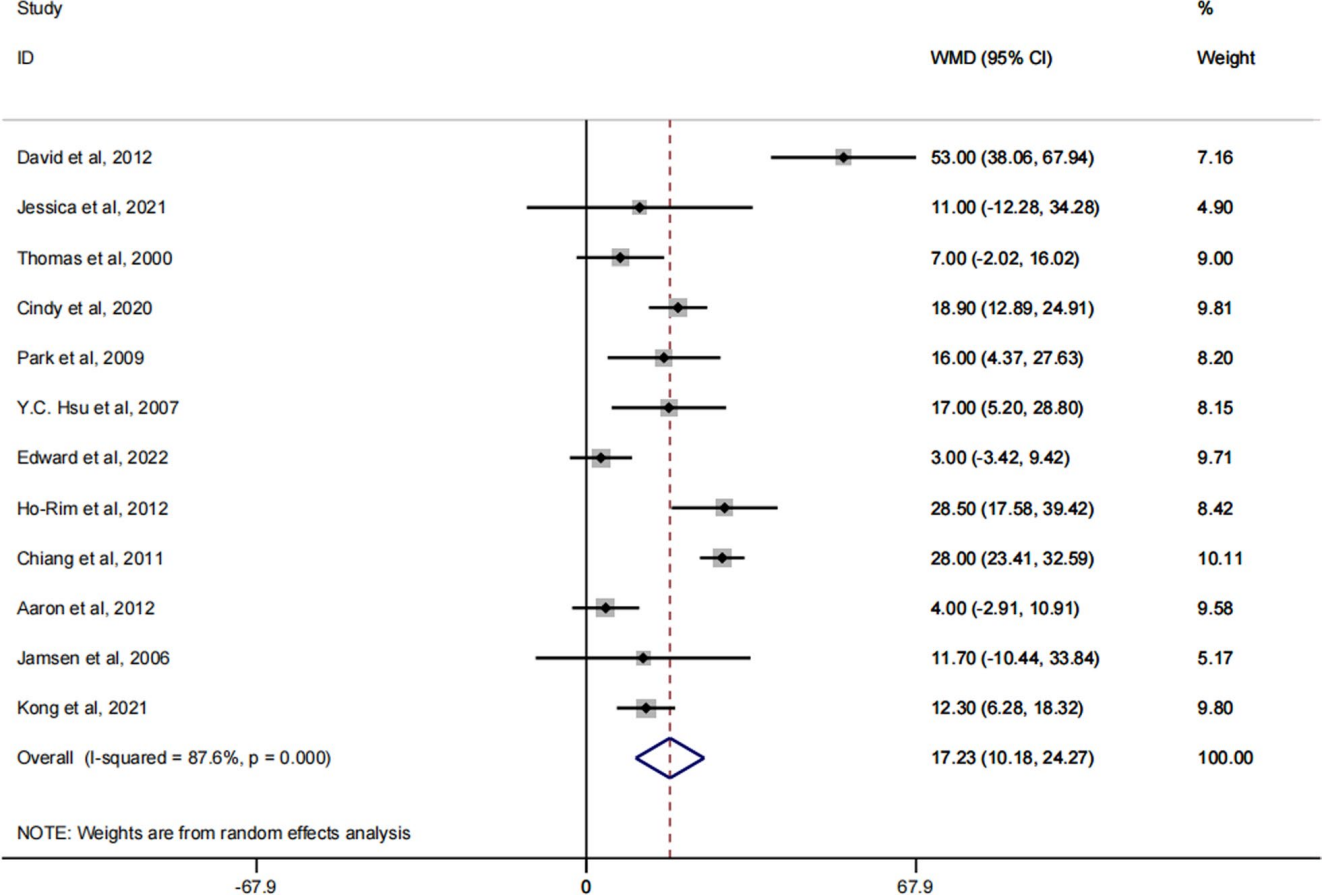
Forest plot of the ROM

A total of 12 of these articles reported on the ROM of the knee joint after patients received spacer implantation [27–35, 37–39]. The result of the meta-analysis suggested to us that the patients with dynamic spacers had significantly better ROM than those with static spacers, (RR: 18.73; 95% CI 11.67, 25.79; P < 0.0001). The specific forest plot is shown in Fig. 7.

## Discussion

With the increase of total knee arthroplasty surgery year by year, the incidence of prosthetic joint infection is also increasing. When a joint prosthesis becomes infected, a poorly controlled infection can lead not only to failure of the joint arthroplasty, but in severe cases even to amputation [40, 41]. Two stage revision surgery is the most commonly used method in clinical practice and it can offer the best results in terms of treatment. In the two stage revision surgery, the commonly used spacers include dynamic spacers and static spacers and each has its own characteristics. The structure of the dynamic spacer is match to the anatomy of the knee joint, thus it can reduce adhesions and scarring of the soft tissues surrounding the knee joint. The dynamic spacer can also improve the recovery of knee function after revision surgery and reduce the incidence of some complications. However, there are also some reports suggesting that dynamic spacers are less effective in controlling prosthetic joint infection. In contrast, static spacers are less prone to dislocation during fixation and it can also provide good joint stability. In addition, the static spacer can provide a high concentration of antibiotics for better infection control while maintaining limb length. Of course, there is still controversy in clinical and related research regarding the difference between dynamic and static spacers in controlling prosthetic infection and improving the prognosis of revision surgery. In the current meta-analysis, we collected relevant clinically controlled studies and performed a meta-analysis of these. Our aim is to further investigate the differences between dynamic spacers and static spacers in terms of therapeutic effect and impact on knee function.

A total of 14 articles were included in this meta-analysis, and we grouped one primary and four secondary outcome indicators according to the indicators in each article. Finally, we did the corresponding meta-analysis for each of the five outcome indicators. The rate of infection control in prosthetic joint infection is the most important indicator of the effectiveness of revision surgery treatment. Based on the final results we can find that the treatment effect between dynamic spacers and static spacers is the same in terms of the main outcome indicator of prosthetic infection control, with no significant difference between the two groups (p = 0.179). This suggests us that dynamic spacers are no less effective than static spacers for prosthetic infection control and it can also achieve the same clinical outcomes. This result is consistent with the results of many current clinical studies and further validates the efficacy of joint spacers [42–44]. According to relevant literature reports, the concentration of antibiotics and the duration of anti-infection therapy are important factors in the outcome of two-stage revision surgery for prosthetic infections. The use of adequate antibiotic concentrations and duration of anti-infection therapy not only results in more satisfactory infection control, but also better reduces the recurrence of prosthetic infections [45, 46]. In addition, revision surgery at an early stage of prosthetic infection can also provide better control of prosthetic infection, and early anti-infection treatment is one of the keys to treatment and reduces the risk of surgery [47, 48]. Therefore, not only does the correct use of spacers will influence the outcome of treatment, but timely and adequate anti-infective treatment is also crucial to the success of treatment.

Of the four secondary outcome indicators, KSSs scores, KSS functional scores and ROM were used to evaluate the impact on knee function after patients received spacer implantation, all as an indicator of post-operative recovery and efficacy. In the results of our meta-analysis, the results for all three indicators were statistically significant (p < 0.05). This indicates a significant difference between the results of the dynamic spacer group and the static spacer group in these three indicators. In terms of improved prognosis for knee function, patients with dynamic spacers had significantly better functional scores and knee mobility than those with static spacers. These results once again verified the superiority of the dynamic spacers over the static spacers, in comparison, the dynamic spacer could better improve the patient's motion function and range of motion [49–51]. Bone loss is a common complication after spacer implantation and the patient's bone loss is a judgment indicator to evaluate the impact of the spacer on the patient's bone health. Therefore, bone loss was also one of the reference indicators in our current meta-analysis. Based on the results of the meta-analysis, it is known that the patients with dynamic spacers experienced significantly less bone loss than those with static spacers (p < 0.05). There was a significant difference in the results between the two groups. This is also in line with some current findings that patients using static spacers are more likely to experience bone loss [52]. Combining these results, we can see that the dynamic spacer can achieve similar results to the static spacer in terms of infection control of the prosthesis. The performance of dynamic spacers is better than that of static spacers in improving the prognosis of knee function and preventing bone loss. For patients requiring prosthetic revision surgery, the use of dynamic spacers may provide a better prognosis and recovery of joint function.

Of course, there are some limitations to our current meta-analysis. Firstly, although we included a total of 14 relevant literatures, the total number of patients studied was only 799, which may not be a large enough sample size. Perhaps we need more clinical studies with larger samples to further confirm our results. Secondly, the articles we included were not clinical randomized controlled studies, but rather retrospective clinical studies and prospective trials. The use of blinding was also not reported for the assessment of outcomes and scores related to knee function, which lacks a certain degree of concealment. In addition, there is a lack of uniformity in the assessment and the associated results may be influenced by subjective factors. Thirdly, the possible influence of relevant factors on the treatment outcomes (e.g., gender, age, height, weight, etc.) was not adequately considered in determining patient groupings.

## Conclusion

Based on the results of the meta-analysis, we finally obtained, we can know that there was no significant difference in the rate of infection control of the prosthesis between the dynamic spacer group and the static spacer group, similar results can be obtained with both types of spacers. And there are significant differences between the dynamic spacer group and the static spacer group in terms of knee function related scores and ROM. The patients using dynamic spacers can achieve better joint mobility and function. There is also a significant difference between the two groups in terms of bone loss. Patients with dynamic spacers experienced significantly less bone loss than those with static spacers.
